# A case of a perforated obturator hernia with a femoral abscess treated a pectineus muscle flap

**DOI:** 10.1186/s12893-015-0095-0

**Published:** 2015-10-02

**Authors:** Naoki Kubo, Zyunichi Yoshizawa, Takaomi Hanaoka, Koichi Nakamura

**Affiliations:** The Department of Surgery, Azumi General Hospital, 3207-1, Ikeda, Ikeda-cho, Kitaazumi-gun, Nagano 399-8695 Japan; The Department of Orthopedics, Azumi General Hospital, 3207-1, Ikeda, Ikeda-cho, Kitaazumi-gun, Nagano 399-8695 Japan

**Keywords:** Obturator hernia, Perforated, Femoral abscess, Pectineus

## Abstract

**Background:**

An obturator hernia accompanied with a femoral abscess is rare, and leads to severe infection. Repeated draining is often required due to remnant abscess.

**Case presentation:**

We herein reported a case of a perforated obturator hernia with a femoral abscess that was successfully treated via repair using the pectineus muscle. An 84-year-old Japanese woman was referred to our hospital with appetite loss and right femoral pain. Abdominal computed tomography (CT) revealed a right obturator hernia and abscess spreading to the right thigh. Emergency surgery was performed. Intraoperative findings revealed that the abscess had formed because of a perforation in the small intestine by an incarcerated obturator hernia. We performed partial resection of the small intestine, repaired the hernial orifice, drained the right femoral abscess, and filled the cavity using the pectineus muscle. A residual abscess was not detectable following surgery, and the patient was discharged on postoperative day 63.

**Conclusion:**

Some patients with a perforated obturator hernia and femoral abscess have a residual abscess following surgery that requires redrainage. Nevertheless, we consider it possible to successfully treat a perforated obturator hernia with a femoral abscess via repair using the pectineus muscle.

## Background

Obturator hernias are rare in acute surgical wards, accounting for only 0.07 % of all hernias, and typically occur in elderly emaciated women as a result of a widened pelvic and enlarged obturator canal [1]. Patients commonly present with symptoms of intestinal obstruction and, occasionally, pain along the distribution of the obturator nerve on the ipsilateral side (Howship-Romberg sign). However, difficulties are associated with an accurate diagnosis, with delays in its diagnosis or surgical interventions contributing directly to the high morbidity and mortality rates that are characteristic of the perforation of gangrenous bowels [2]. An obturator hernia accompanied by a femoral abscess followed by perforation is a very rare combination, but may lead to severe infection and takes time to completely heal. We herein reported a case of a perforated obturator hernia with a femoral abscess that was successfully treated via repair using the pectineus muscle.

## Case presentation

An 84-year-old Japanese woman was referred to our hospital with appetite loss and vomiting. She saw her family doctor three weeks previously, but was only diagnosed with appetite loss. A physical examination showed that her vitals were stable, but that she was dehydrated. Abdominal distention was observed, and palpation revealed a hard mass in the medial side of the right thigh that was tender. Her white blood cell count was 13,060/ml, C-reactive protein level was 11.5 mg/dl, and temperature was 38 °C.

**Fig. 1:**
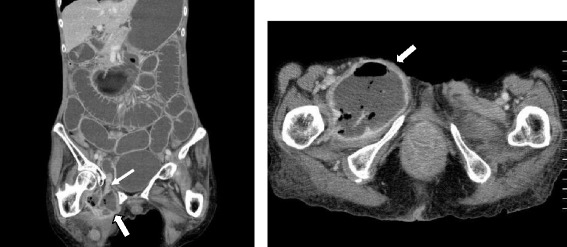
Computed tomography (CT) revealed a right obturator hernia (*thin arrow*) and abscess spreading to the right thigh (*bold arrow*)

**Fig. 2:**
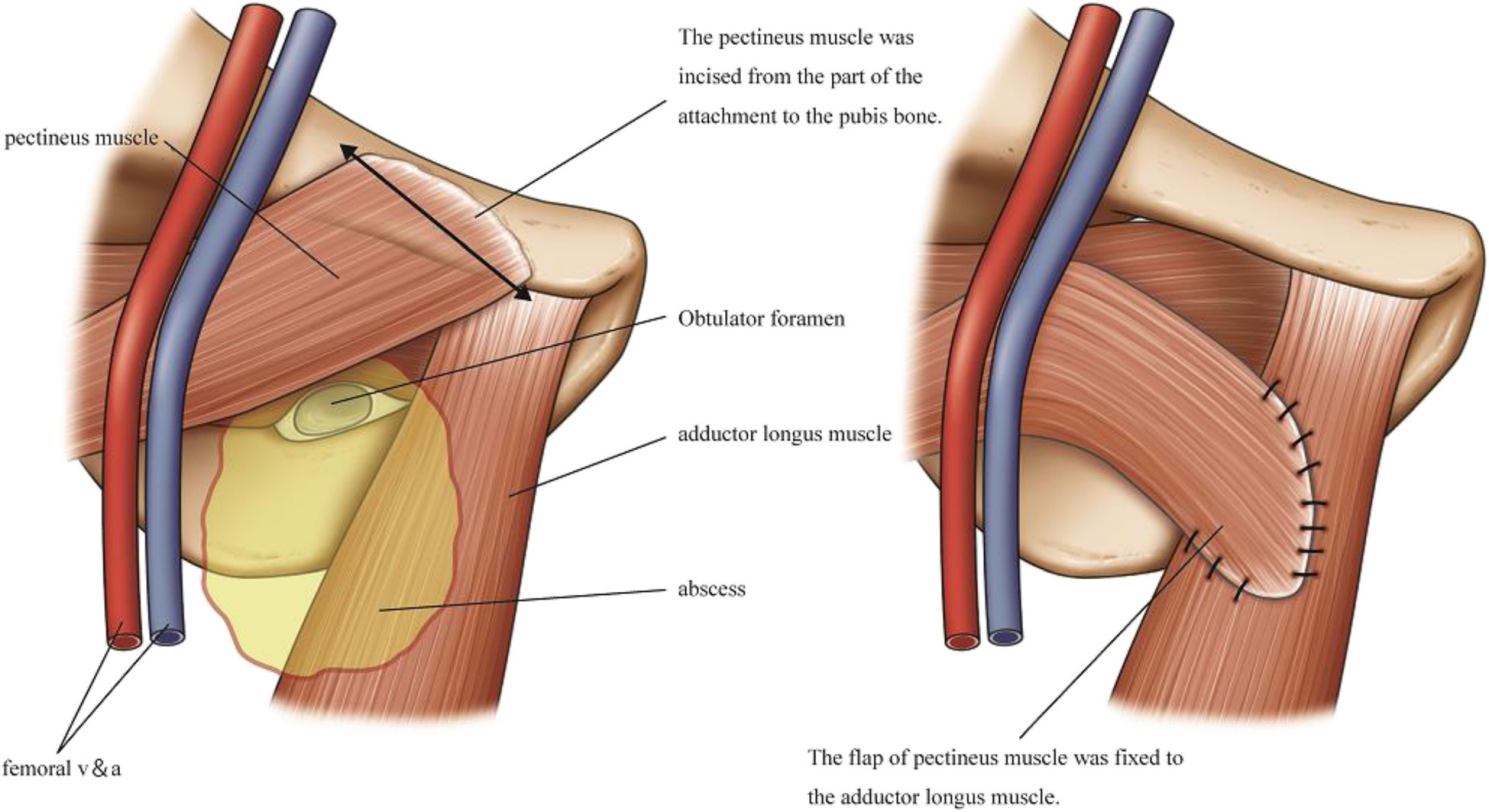
Schema of the pectineus muscle flap

**Fig. 3:**
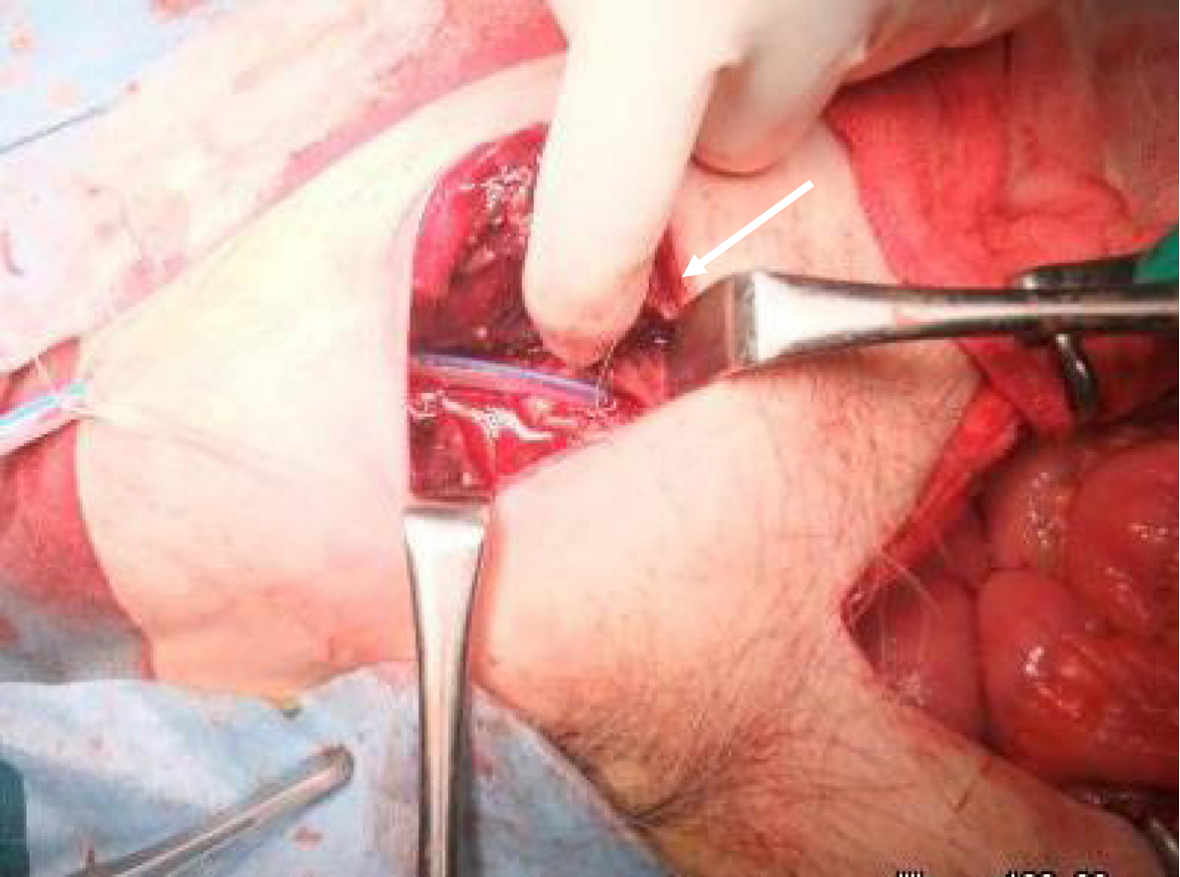
Intra-operative image of the femoral abscess after incision and drainage (*thin arrow*)

Abdominal computed tomography (CT) showed a proximal small bowel obstruction secondary to the right obturator hernia as well as an abscess spreading to the right thigh (Fig. 1). We determined that the abscess had formed because of a perforation in the small intestine by an incarcerated obturator hernia; therefore, emergency surgery was performed. During laparotomy of the lower median, the small intestine was revealed to be herniated into the right obturator canal. The proximal intestine was severely dilated and the distal intestine had collapsed. The incarcerated intestine was reduced, and the abscess had formed due to a perforation in the small intestine by the incarcerated obturator hernia. We performed partial resection of the small intestine and simple closure of the obturator foramen. A large amount of pus was drained at incision of the right femoral below the inguinal ligament and on the inside of the femoral artery, and sufficient irrigation was performed. We repaired this site by filling the drained femoral abscess with the pectineus muscle, which had been incised from part of the attachment to the pubis bone, and fixed it to the adductor longus muscle in order to reduce the residual abscess and risk of recurrence after surgery (Fig. 2). Two 15 French continuous suction drains (J-VAC®) were placed in the thigh (Fig. 3), and the skin was closed. A residual abscess was not detectable following surgery, and the drains were removed on postoperative day 12.

Although the patient had previously been reliant, she had become bedridden prior to emergency surgery until approximately two weeks after surgery. She subsequently underwent rehabilitation and was gradually ambulatory one month after surgery. She was discharged on postoperative day 63.

## Discussion

Hernial orifices in obturator hernias are small and inflexible; therefore, incarcerated Richter’s hernias commonly occur. Thus, any delay in a diagnosis may lead to necrosis and perforation of the small bowel or a concomitant femoral abscess, as was observed in the patient in the present case, and peritonitis. However, recent advances in diagnostic imaging have led to improvements in the diagnosis rate of obturator hernias using computed tomography (CT) and other imaging techniques [3–5]. The only treatment for obturator hernias is surgery. Various surgical approaches include abdominal, inguinal, retropubic, and transperitoneal and, more recently, laparoscopic approaches. Simple closure of the hernia defect with interrupted sutures or placement of a synthetic mesh are the preferred method of herniorrhaphy because they are associated with the lowest complication rates [3, 4].

An obturator hernia with a concomitant femoral abscess is extremely rare, with only 16 cases (including the present case) being reported to date in Japan [6]. The average age of patients in these reports was 83.7 years, and all patients, except for one, were female. Since the average number of days between disease onset and surgery was approximately 19, delays in diagnoses were implicated in the formation of abscesses.

The treatment of an obturator hernia with a concomitant femoral abscess requires resection of the perforated section of the small bowel and drainage of the femoral abscess. In some cases, the femoral abscess is opened first and laparotomy and resection of the perforated section of the small bowel are performed at a later date in a second surgical procedure. However, in other cases, resection of the perforated section of the small bowel and drainage of the femoral abscess are both performed in a single surgical procedure. In cases reported in Japan, four patients were treated with the two-stage surgical approach and 12 patients were treated with the single-stage surgical approach. It was previously impossible to diagnose an obturator hernia as the cause of a femoral abscess prior to surgery, which led to treatments using the two-stage surgical approach, because, after incisional drainage of the femoral abscess was performed, the leakage of intestinal fluid and findings of a subsequent fistulogram finally led to the diagnosis of an obturator hernia. Recent advances in the use of CT and other imaging methods have led to an increase in the early diagnosis of obturator hernias; therefore, most patients now undergo single-stage surgery. Two-stage surgery is performed in serious cases; the femoral abscess is first drained and inflammation is reduced to minimize risk, and this is followed by intestinal resection. On the other hand, in most cases (7 out of 10) in which the thigh wound was closed during a single-stage procedure, the wound had to be reopened later to allow drainage. In spite of this drainage, disseminated intravascular coagulation (DIC), necrotizing fasciitis, and methicillin-resistant Staphylococcus aureus (MRSA) infection were detected after surgery in four cases in which the thigh wound was closed, and two of these patients died. The treatment of a femoral abscess requires adjustments to the surgical technique used in order to allow sufficient drainage from the narrow obturator canal and small intermuscular gaps and also to avoid leaving a remnant abscess. In some cases, the wound is not closed after drainage, which is an option that requires consideration of the patient’s general condition. However, wounds left open require more time to heal, which may decrease activities of daily living (ADL) in elderly patients. In order to avoid this, muscle flaps are sometimes used to close refractory abscesses. Previous studies in the field of gastrointestinal surgery reported relatively good wound healing when using a surgical procedure that involved filling abscess cavities in the pelvis with the gluteus maximus muscle after rectal amputation [7, 8]. In the present case, we prevented the recurrence of the abscess by filling the cavity with the pectineus muscle, which was easily accessible from the same surgical field. No remnant abscess was detected after surgery, and we were able to remove the drain relatively soon after surgery, which allowed us to recommend that the patient mobilize as soon as possible. Using the pectineus muscle as a filler is a method that is useful in cases of an obturator hernia with a concomitant femoral abscess.

## Conclusion

A delay in the diagnosis of an obturator hernia may lead to intestinal necrosis and a femoral abscess. Although a femoral abscess is a rare complication in obturator hernia cases, insufficient drainage has been reported to lead to necrotizing fasciitis and other serious infections [9–11]. Since repeated draining is often required due to remnant abscesses, adjustments to drainage methods and measures to prevent remnant abscesses are required. We herein reported a case in which a satisfactory outcome was achieved by using the pectineus muscle as a filler in the treatment of an obturator hernia that caused a femoral abscess.

## Consent

Written informed consent was obtained from the patient for publication of this Case report and any accompanying images. A copy of the written consent is available for review by the Editor-in-Chief of this journal.
